# Intensifying responsiveness towards neglected intestinal helminth infections in a resource-constrained setting

**DOI:** 10.1186/s41182-017-0053-x

**Published:** 2017-05-08

**Authors:** Khin Thet Wai, Kay Thwe Han, Tin Oo

**Affiliations:** Department of Medical Research, No. 5 Ziwaka road, Yangon, 11191 Myanmar

**Keywords:** Neglected intestinal helminth infections, Resource-constrained settings, Transmission elimination plan, Research gaps

## Abstract

Neglected intestinal helminth infections afflict the marginalized communities in Asia. Since 2004, growing body of evidence in Myanmar indicated high prevalence of soil-transmitted helminths (STH) infections (30–40%) among school children. Co-existence of STH (23%) with food-borne trematodes was noted among 383 pregnant women in a selected township in 2012–2014 followed by molecular verification of very low prevalence of schistosome infection (<5%) in the same study site in 2016. The success of transmission elimination plans may depend upon sensitive diagnostic tools to detect persistent infections and polyparasitism. Addressing the research gaps in vulnerable sites requires an increased investment in resource-constrained settings.

## Background

Neglected intestinal helminth infections such as soil-transmitted helminths (STH) afflict the marginalized communities in Asia including Myanmar. Globally, World Health Organization (WHO) calls for scaling up interventions by improved coverage in regular deworming of preschool and school-aged children from 50 to 75% by 2020 [1, 2]. Earlier studies in Myanmar provided an evidence of negative impact of STH on community health and the effectiveness of deworming on the nutritional status of children [3]. Since 2004, the growing body of scientific evidence in Myanmar indicated the high prevalence of STH infections (30–40%) among school children in the central plains and coastal region [4]. Following the membership of the Regional Network on Asian schistosomiasis and zoonotic helminths (RNAS+) in 2013, scientists from Department of Medical Research, Myanmar further explored the vulnerable ecosocial context for zoonotic helminth infections in freshwater wetlands of Myanmar [5]. The coexistence of STH (23%) with food-borne trematodes (FBT) was noted among 383 pregnant women in Shwegyin township, Bago Region that covered villages along the riverside, inland, and hilly areas [6]. Helminth infections could exacerbate anemia in pregnancy in malaria endemic areas as in the cited study.

Recently published research work addressed detection of schistosome antibodies with ELISA in for the first time in the natural lake site at the hilly region of Myanmar in 2014. This study pointed out the necessity for further parasitological confirmation due to false positive cross-reactions [7]. In contrast, the subsequent study in the lower part of Myanmar where there was a mix of ecological context (riverside, inland, and hilly areas), 204 stool samples were examined first by conventional Kato-Katz technique for detection of helminth eggs [8]. Out of 31 stool samples microscopically positive for at least one helminth infection, 8 samples (3.9%) were verified by conventional polymerase chain reaction (PCR) as *Schistosoma Mekongi* infection. Further confirmation was made in National Institute of Parasitic Diseases, Chinese CDC, Shanghai through South-South collaboration during 2016. Very low prevalence of schistosome infection (<5%) was not linked to any form of intervention in that particular study site for example improved water, sanitation, and hygiene interventions.

Thus, application of molecular techniques could verify the Schistosoma infection as *S. Mekongi* which has never been reported in Myanmar. The findings also highlighted the role of advanced molecular techniques to detect the neglected, unexplored, burden of zoonotic and food-borne disease. Myanmar in a resource-limited setting, multidirectional, regional, and extra-regional collaborations might benefit to address this important research gap in support of decisions for control and elimination. The piecemeal of evidence of schistosomiasis in Myanmar required further confirmation of local transmission while moving towards transmission elimination of multiple helminth infections (polyparasitism).

## Investment for advanced diagnostic techniques and deworming

Kato-Katz (K-K) technique is the classical parasitological investigation which is based on the microscopic observation of the parasite eggs that could bring about 100% specificity. However, in rare conditions such as schistosomiasis in low endemic settings without any proven evidence, sophisticated molecular techniques might provoke an answer for species identification as an adjunct to K-K. We need to invest for advanced diagnostic techniques to conduct the initial nationwide survey in support of transmission elimination of multiple helminths apart from the conventional K-K technique. These tools will be able to detect broader array and intensity of helminthic infections compared to conventional method. An investment is necessary for introducing new diagnostic tools for valid and reliable age-specific prevalence estimates and ascertaining the modifiable risk factors, potential parasite reservoirs, and persistence of poly parasitism. Apparently, appropriate use of conventional techniques and advanced molecular techniques are desirable from a cost-benefit viewpoint. Sensitive tools may detect non-specific results when stool samples are applied because many unidentified molecules exist in stool. On the other hand, tools using expensive reagents are questionable for mass screening. The elucidation of heterogeneity in multiple helminth infections including schistosomes among ecologically vulnerable populations (children, pregnant women, migrant workers, and native villagers) in resource-constrained settings like Myanmar might support the recent call to strengthen the national as well as global strategies against schistosomiasis and STH [9] leading towards an integrated control.

Misleading public health advocacy and decisions could stem from incorrect predictions and wrong estimates in co-endemicity of disease burden. Although we are working towards getting an investment to correct research gaps and to translate evidence into action, control programs also need to pay attention to a lifestyle of people in Myanmar. The importance of accelerating efforts in large-scale treatment programs towards reducing the incidence of STH, FBT, and schistosomes are well documented. Montresor et al. in 2004 [10] revealed crude calculations in estimated cost for helminth control in Myanmar during the pilot exercise that covered 25,000 school children in 200 schools as approximately 0.05 USD per child. Further analyzing the cost-effectiveness of current STH control programs in recent years in line with changing economy may support evidence-based decisions of program planners. In areas where these infections overlap, WHO recommended for large-scale treatment of both diseases to at-risk populations given the prevalence of STH as 20% and beyond, and the prevalence of schistosomiasis as 10% and beyond [11]. Given that additional control mechanisms are required in Myanmar to commensurate with an increase in total population up to 51.4 million according to National Census 2014, an increased investment in mass deworming is desirable for township level health system strengthening. Moreover, program planners need to consider the population growth rate, urbanization, school-based deworming programs, national filariasis elimination strategies, nationwide coverage of improved water supply, sanitation and hygiene programs, and climate change and natural disasters.

To date, the information on prevalence and risk factors of schistosomes and FBT concurrently with other helminth infections is limited in Myanmar. As emphasized by Disease Reference Group 4, WHO-TDR and STH control in Myanmar reaches the stage of infection control, while other helminth infections that include schistosomiasis, food-borne trematodiasis, and cysticercosis/taeniasis are only at the initial stage of exploring the extent of morbidity [11]. Undocumented evidences and questions remain in the context of limited resources that could pave the way to global elimination of neglected intestinal helminth infections by 2020 [12, 13]. The changing governance structure and undergoing major health system reforms focus on demand and supply side issues in impoverished and vulnerable sites of Myanmar. Research gaps mean the discrepancy between knowledge of the prevalence of helminth infections and evidence-based deworming plans. Those gaps stem from discrepancy between the knowledge of program planners and the real morbidity of the area where the control program is implemented. Research gaps to ascertain the magnitude of the problem of schistosomiasis and FBT apart from STH mainly stem from the hidden problem, left undiagnosed, untreated, and under-reported in the past decade. Due to an increase in population mobility in recent years as well as climate change and natural disasters, there might be changes in locations of intermediate hosts and human reservoirs. However, the pivotal role of research in disease control programs especially concerning with neglected intestinal helminth infections is not well recognized as important in resource-constrained settings.

Thus, gaps for research priorities in Myanmar and in other resource-constrained settings to support transmission elimination of intestinal helminth infections in vulnerable age-groups require identification of morbidity at the sub-national level, township level and below to alleviate inequities, to strengthen primary health care, and for poverty reduction and area development. A blueprint for success is to integrate control of STH and other intestinal helminths with Disease Control Programs apart from Maternal and Child Health, Nutrition and School Health Programs for widespread coverage and recognition in resource-limited settings.

## Conclusions

Evidence-based control mechanisms by addressing the research gaps in vulnerable sites require an increased investment in resource constrained settings. In a changing health policy landscape and health scenario in Myanmar, major reforms focus impoverished sites. In the years to come, improved surveillance and transmission elimination strategies of STH and FBT in Myanmar among at-risk populations needs an implementation research to overcome challenges leading to sustained control and to prioritize pragmatic solutions for high impact. Moreover, it is crucial to increase an investment to conduct research on standardization and validation of diagnostic techniques in support of monitoring and evaluation and epidemiological surveillance and to develop and evaluate innovative awareness raising strategies among risk groups in endemic regions of resource-constrained settings.
